# Glutamate in Salience Network Predicts BOLD Response in Default Mode Network During Salience Processing

**DOI:** 10.3389/fnbeh.2019.00232

**Published:** 2019-10-02

**Authors:** Felicia von Düring, Inka Ristow, Meng Li, Dominik Denzel, Lejla Colic, Liliana Ramona Demenescu, Shijia Li, Viola Borchardt, Thomas Liebe, Matthias Vogel, Martin Walter

**Affiliations:** ^1^Clinical Affective Neuroimaging Laboratory, Otto von Guericke University Magdeburg, Magdeburg, Germany; ^2^Max Planck Institute for Biological Cybernetics, Tübingen, Germany; ^3^Department of Behavioral Neurology, Leibniz Institute for Neurobiology, Magdeburg, Germany; ^4^Department of Psychiatry, Yale School of Medicine, New Haven, CT, United States; ^5^School of Psychology and Cognitive Science, East China Normal University, Shanghai, China; ^6^Department of Psychosomatic Medicine and Psychotherapy, Otto von Guericke University Magdeburg, Magdeburg, Germany; ^7^Department of Psychiatry and Psychotherapy, Otto von Guericke University Magdeburg, Magdeburg, Germany; ^8^Center for Behavioral Brain Sciences, Magdeburg, Germany; ^9^Department of Psychiatry and Psychotherapy, University of Tübingen, Tübingen, Germany; ^10^Department of Psychiatry and Psychotherapy, University of Jena, Jena, Germany

**Keywords:** PCC, fMRI, MRS, glutamate, salience attribution, attention

## Abstract

**Background:**

Brain investigations identified salience network (SN) comprising the dorsal Anterior Cingulate Cortex (dACC) and the Anterior Insula (AI). Magnetic resonance spectroscopy (MRS) studies revealed the link between the glutamate concentration in the ACC and alterations in attentional scope. Hence, we investigated whether glutamate concentration in the dACC modulates brain response during salience processing.

**Methods:**

Twenty-seven healthy subjects (12♀, 15*♁*) provided both STEAM MRS at 7T measuring glutamate concentrations in the dACC as well as a functional magnetic resonance imaging (fMRI) task to study the influence on content-related salience processing and expectedness. Salience was modulated for both sexual and non-sexual emotional photos in either expected or unexpected situations. Correlation between MRS and task fMRI was investigated by performing regression analyses controlling for age, gender, and gray matter partial volume.

**Results/Conclusion:**

During picture processing, the extent of deactivation in the Posterior Cingulate Cortex (PCC) was attenuated by two different salience attributions: sexual content and unexpectedness of emotional content. Our results indicate that stimulus inherent salience induces an attenuation of the deactivation in PCC, which is in turn balanced by higher level of glutamate in the dACC.

## Introduction

Evaluation of salient information dominates our daily life.

High salient stimuli, for instance an erotic photo on a public advertisement, automatically capture our attention by standing out relative to their environment and containing intense visual input. Next to such stimulus inherent properties, the actual content of presentation in either an expected or unexpected situation drives the amount of attention allocated to a stimulus.

The so-called salience network (SN) consists of paralimbic anterior cingulate and frontoinsular cortices and shows broad connectivity to subcortical and limbic structures. Key regions are anchored in the dorsal Anterior Cingulate Cortex (dACC) and the Anterior Insula (AI). It is involved in perception of salience and guides switching between externally and internally focused attention (Menon and Uddin, 2010).

The SN interacts with other large-scale brain networks, such as the default mode network (DMN) consisting of PCC, ventromedial Prefrontal Cortex, lateral Inferior Parietal Lobe, and medial temporal structures (Greicius et al., 2009), to orchestrate behavior upon certain cognitive constraints (Menon, 2011). It is commonly known that the DMN reduces its activity gradually when other networks show increased activity. For example, the DMN’s activity decreases upon increasing difficulty of working memory tasks and takes several minutes to return to a stable baseline level (Barnes et al., 2009; Vatansever et al., 2015; Fan et al., 2019).

Prior functional connectivity analyses showed that differential network integration of the SN influences character traits such as novelty seeking (Li et al., 2017; Rueter et al., 2018) and susceptibility to distraction (Götting et al., 2017). For example, resting state connectivity between the SN and the PCC (part of DMN) correlated with the susceptibility to salience interference. This indicates that the interaction of PCC and dACC plays a role in attentional focusing toward subjectively relevant information (Götting et al., 2017). Indeed, studies specified the role of the PCC in modification of attentional focus (Bush et al., 2000; Leech and Sharp, 2013). Leech and Sharp (2013) highlighted that the PCC acts as a hub facilitating integration across multiple networks and elaborated the role of the PCC during attention toward internal and external targets, considering the breadth of attentional scope. Fan et al. (2019) described the interaction between the dorsal and ventral PCC subregions and their differential involvements in regulating environmental demands. To be specific, the ventral portion of the PCC is considered to be a core node interplaying with the DMN whereas the dorsal PCC has a high functional connectivity to the central executive network (CEN) at rest, as well as both during and following affective processing. The dorsal PCC furthermore orchestrates switches of between network connectivity patterns during affective and cognitive task execution (Fan et al., 2019).

The Cingulate Cortex (CC) is involved in a broad range of processes involving specific adjacent subregions. Therefore, Dou et al. (2013) segregated the region into four subregions namely pregenual ACC (pgACC), dACC as well as rostral and caudal CC showing significantly different mean levels of several metabolites such as glutamate. The role of the most abundant excitatory neurotransmitter – glutamate measured in the dACC, was investigated by combined MRS/fMRI studies in the past. Falkenberg et al. (2012) showed that interindividual variations of glutamate levels in the dACC, a core of the SN, influence BOLD activations in the DMN depending on cognitive load. The authors identified glutamate to be involved in the interaction between the task-dependent BOLD response and DMN by contributing to (de)activation of these networks. Furthermore, glutamate exhibits a global effect on BOLD response via glutamatergic projections to other cortical regions rather than modulating the BOLD response within the acquired MRS voxel (Falkenberg et al., 2012; Duncan et al., 2014). Based on these findings, the authors suggested a more global effect of glutamate on BOLD response, whereas they indicated that GABA might be more responsible for local BOLD effects.

To contribute to the understanding of interindividual differences reflected in brain activity and connectivity, MR spectroscopy studies showed that local glutamate concentration in the ACC predicts ACC BOLD activity (Enzi et al., 2012). Data has suggested a metabolic effect on between (Horn et al., 2010; Demenescu et al., 2017) and within network connectivity (Kapogiannis et al., 2013), as well as properties of routing efficiency of whole brain integration using graph analysis (Lord et al., 2017). To be more specific, Horn et al. (2010) for instance, reported that connectivity between the pgACC and the Insula correlate with glutamate levels measured in the pgACC but not the Insula.

Prior studies that have investigated ACC subregions, provided evidence for receptor-architectonical and functional segregation (Palomero-Gallagher et al., 2009) consequently causing significantly different regional distribution of metabolite concentrations, namely inhibitory neurotransmitter GABA, excitatory glutamate and their precursor glutamine. For example, significantly higher GABA and glutamate concentrations were found in the pgACC as compared to other three cingulate subregions (Dou et al., 2013).

The effect of varying glutamate levels and DMN-SN connectivity was furthermore investigated in psychiatric patients. In clinical samples with depression or ADHD, abnormal connectivity between e.g., the dACC and the PCC was discussed in the context of local cellular aberrations (Castellanos et al., 2008; Zhang et al., 2016) and the association between the ACC neurotransmitter levels and activity and functional connectivity was hinted to reflect severity of abnormal salience mapping (Walter et al., 2009; Horn et al., 2010).

These studies corroborate the assumption that neurometabolites modulate functional connectivity and activity. In our study when using the term “modulation” we want to express that the SN influences differential activations/responses toward conditions of different salience. The influence was operationalized in terms of between subject co-variation of the actual BOLD contrasts and local glutamate concentrations. This modulation was assumed since measured glutamate and observed BOLD contrasts would not have to appear in the same region. As it has been postulated before, glutamate exhibits a modulating effect on remote activity via long range projections from glutamatergic excitatory neurons (Falkenberg et al., 2012; Duncan et al., 2014). What is missing to date, is an investigation if metabolite concentrations in the core region of SN indeed modulate differential activity on a whole brain level, which itself is classically associated with salience processing.

Salience, for example, can be introduced contextually by different levels of expectedness (Bermpohl et al., 2006) or by stimulus inherent properties. These aspects can be further combined in tasks which present highly salient erotic or low salient emotional stimuli in either expected or unexpected situations (Walter et al., 2008) and thus allow for an investigation of the modulation by both mechanisms in the same paradigm (Metzger et al., 2010). Walter et al. (2008) reported that in a medial prefrontal DMN region, deactivations were smaller for erotic pictures with increasing positive valence, which were then considered more sexually salient. Importantly, for both anterior and posterior DMN regions, attenuation of deactivation for individual trials was directly correlated with the individually perceived sexual intensity of each stimulus. While ventral medial Prefrontal Cortex (MPFC) effects were discussed to be modulated by an interaction of sexual intensity and valence, dorsal MPFC and Precuneus effects were related to the modulation of deactivation as a function of general emotional arousal (Walter et al., 2008). The authors discussed this effect as a residual attentional focus on elicited subjective feelings, which increases during emotionally salient stimulation and thus limits the externalization of the attentional focus.

This type of paradigm, next to activating the dACC during expectancy cue periods, also leads to differential activations of the DMN in subsequent picture periods as a function of salience (Bermpohl et al., 2006; Walter et al., 2008).

Using such combined salience task, we expected an influence of glutamate measured in the SN on salience dependent differential (de)activation in the DMN, thus reflecting a remote effect of differential influence of SN on brain response integration. The glutamate levels were examined *post hoc* in relation to the significant clusters from the whole-brain analysis.

## Materials and Methods

### Participants

Forty healthy subjects (mean age ± standard deviation (sd) = 29.82 ± 7.84 SD, 14 women) were recruited via local advertisement. Absence of psychiatric and neurological diseases according to ICD-10 criteria (Dilling and Freyberger, 2012) was ensured by examination and interview by the study physician. During the interview subjects completed Mini International Interview (MINI) (German version, Ackenheil et al., 1999), Young Mania Rating Scale (YMRS) (Young et al., 1978) and Hamilton Depression Scale (HAM-D) (Hamilton, 1986). All subjects were right-handed as assessed with the short version of the Edinburgh Handedness Inventory (Oldfield, 1971). The institutional review board of Otto von Guericke University Magdeburg approved the study and all participants provided written informed consent.

### Anatomical Images

MR data were obtained between 12 am and 5 pm on a 7T scanner with a 32 channel head array coil (Siemens Healthineers, Erlangen, Germany). Automated global shim was performed. Using a magnetization prepared - rapid gradient echo (MPRAGE) sequence (TE = 2.73 ms, TR = 2300 ms, TI = 1050 ms, flip angle = 5°, bandwidth = 150 Hz/pixel, isotropic voxel size = 0.8 mm) high resolution T1–weighted anatomical MR images were acquired first. Anatomical images were segmented (VBM8 in SPM8^1^) and used for co-registration of functional data and for calculating subject-based gray matter partial volume for the dACC voxel.

### Magnetic Resonance Spectroscopy Data Acquisition

Before undergoing the fMRI task, glutamate concentration in the dACC was measured via MRS. Automated region – specific shimming was applied using a double-gradient echo shim technique to optimize field homogeneity. Single MRS spectra were acquired at rest in bilateral dACC (25 × 15 × 10 mm^3^ = 3.75 ml). The voxel position was set bilateral and followed the sagittal median line to get maximal coverage of the gray matter (gm) tissue. Stimulated echo acquisition mode sequence (STEAM) was used for the MRS data acquisition, with the following parameters: TR = 3000 ms, TE = 20 ms, TM = 10 ms, 128 averages. A water reference scan was acquired to serve as an internal concentration reference for absolute quantification and also for compensation of eddy currents.

For spectra analysis, the LCModel fitting software (Provencher, 1993) with a stimulate basis set (in total 19 different metabolites) was utilized. The following exclusion criteria were set to ensure good MRS data quality: (I) full-width half-maximal (FWHM) > 24 Hz, (II) signal-to-noise ratio (SNR) < 20, and (III) Cramér-Rao Lower Bound (CRLB) > 20%. Since creatine (total, tCr) is a stable metabolite across subjects in normal but also multiple pathologic states, glutamate levels are reported as their relative amount to total creatine (Glu/tCr).

### Task fMRI

Participants completed an event related expectancy paradigm with positive emotional and erotic pictures (total duration 14 min) (Supplementary Figure S1), which was displayed via New projector JVC DLR-RS49E and a coil-mounted mirror in the scanner. Subjects saw a set of 40 high salient erotic or low salient emotional pictures (stimulus presentation for 3–5 s, divided with fixation cross 7.5–10.5 s, fixed randomized order). Presentation software package (Neurobehavioral Systems^2^) was used to design and run the experiment. Pictures were taken from the standardized picture set – International Affective Picture System (Bradley and Lang, 2007). Two different categories of expectancy cues were applied in the experimental design (Supplementary Figure S1): white centered arrows on a black background indicated the specific content of the subsequent picture. An upward directed arrow announced a non-sexual emotional content of the picture whereas a downward directed arrow depicted an erotic content in the following photo. Second, expectancy category was illustrated by centered white dots on a black background with an adjacent exclamation mark revealing the number of persons appearing in the next photo. The participants knew beforehand whether one (one dot) or two (two dots) persons would appear on the subsequent picture but were not aware of the picture′s content (erotic or emotional). This category was used to circumvent a surprise effect of a completely unexpected picture: the participants actively expect a picture (one person or two persons) but the content itself is unexpected. A fixation cross was presented between the stimuli and served as a break to get rid of all thoughts and expectations of the previous photo. Participants were asked to actively expect the subsequent photo depending on the expectancy cue and then view the photos (passively). All pictures had a preceding cue and there were no negative pictures in the paradigm.

During task, functional single-shot echo planar imaging (EPI) volumes with blood oxygenation level dependent (BOLD) contrast were acquired. The parameters for this measurement were: TE = 22 ms, TR = 2800 ms, flip angle = 80°, volumes = 300, slices = 56, slice thickness = 2 mm, voxel size = 2 mm^3^. On-line motion correction was also applied (Speck et al., 2008).

### Image Preprocessing

Functional EPI and anatomical images were visually inspected to ensure good image quality and to exclude incidental clinical findings. Preprocessing steps included slice timing, realignment, segmentation, co-registration, normalization and smoothing. Here, in order to improve the co-registration accuracy between subjective anatomical image and the EPIs, the segmented native gray and white matter and cerebrospinal fluid (CSF) were combined to a subjective anatomical image. Functional images were then co-registered to this generated T1 image. Lastly, smoothing was performed with a 6 mm full-width-at-half-maximum Gaussian kernel. Image preprocessing was carried out using SPM8 software package running in MATLAB R2013b.

### Statistical Analysis

Different regressors of interest were modeled at a single subject level and convolved with the canonical hemodynamic response function (HRF) based on the general linear model (GLM) embedded in SPM8. Motion parameters were included as regressors of no interest. Design matrix representing different expectancy (X) and perception conditions was created and fixation (fix), Xemo (expectancy of a low salient emotional content), Xamb (expectancy of one or two persons), xPemo (pictures with expected low salient emotional content), xPsex (pictures with expected high salient erotic content), uPemo (pictures with unexpected low salient emotional content), and uPsex (pictures with unexpected high salient erotic content) were implemented.

The primary contrast of interest was (high and low) salient picture viewing versus fixation (*P* > fix), for which one sample *t*-test was run, controlling for age and gender. For the same contrast regression analysis was done with Glu/tCr controlling for age, gender and gm voxel content. Whole brain level analyses were carried out, with a statistical significance set at *p* < 0.05, FWE cluster level corrected, with an initial search threshold of *p* < 0.001, uncorrected. This step was implemented in response to prior criticism based on inflated false positives when using less-stringent initial height thresholds (Eklund et al., 2016). SPM Anatomy Toolbox was used to identify anatomical name of resulting clusters (Eickhoff et al., 2005). Next, to assess the influence of salience – content and expectedness, a ROI analysis was conducted, based on a correlation cluster in the PCC. The choice of subsequent correlations analysis of individual PCC deactivation in the respective task components (salience conditions) was justified by the fact that the PCC was the only region, where both a significant deactivation and a significant correlation of differential activation was observed. Regarding the reported lateral parietal cortices (also part of the DMN) cluster for the task activation (*P* > fix) there was no overlapping cluster between task activation and correlation with glutamate. Consequently the analysis was limited to the PCC cluster as no other cluster from DMN was found in our study. Additionally, residualized Glu/tCr outliers were identified with a box plot in SPSS (<Q1 or >Q3) (IBM SPSS Statistics 20) and the analyses were redone without the potential outliers.

For each picture condition versus fixation mean beta estimates from the above mentioned cluster were extracted (MarsBaR, Marseille, France), and correlated with the Glu/tCr levels in dACC, residualized for age, gender and gm partial volume, in SPSS (with Spearman or Pearson correlation). Correlation slopes were tested for significant difference with Steiger’s test and plotted for display purposes.

Psex > Pemo represents a contrast focusing on differential salience being either sexual (Psex = sexual picture = high salient stimuli) or emotional (Pemo = emotional picture = low salient stimuli) without considering expectedness (whether the stimulus is presented as an expected or unexpected stimulus during task). The contrast uPemo > xPemo examines low salient emotional pictures and the expectedness condition when the stimuli were either presented as an expected (xPemo) or an unexpected (uPemo) picture content. Likewise, the third contrast tests sexual content pictures only and their respective expected (xPsex) and unexpected (uPsex) picture content conditions.

To confirm ROI findings and to set their relevance into context of whole brain mechanisms, we performed additional whole brain level regression analyses (SPM8, *p* < 0.05, FWE corrected, initial threshold *p* < 0.001, uncorrected) of the influence of the Glu/tCr concentration in the dACC on differential picture condition activations by using high salient sexual pictures versus low salient emotional picture (Psex > Pemo) and their subconditions with focus on expectedness such as expected (xPemo) and unexpected (uPemo) emotional pictures (e.g., xPemo > uPemo).

## Results

### Demographic Data

In total, twenty-seven subjects (mean age ± standard deviation (sd) = 29.85 ± 8.26 SD, 14 women) were included into the regression analysis. Thirty-one subjects (mean age ± sd = 29.58 ± 7.86, 14 women) out of the healthy control population (*n* = 40) were included into the final sample for the statistical first level and task activation analysis. In total nine participants were excluded due to task recording issues (*n* = 8) and incomplete task performance (*n* = 1). Furthermore, four subjects did not fulfill the sufficient FWMH, SNR, and SD criteria for MRS measurements (consequently *n* = 27 were included into regression analysis).

### Salient Picture Viewing and Varying Glutamate Concentration in the dACC

#### Salient Picture Viewing Versus Fixation Contrast Revealed Deactivation of the PCC

**FIGURE 1:**
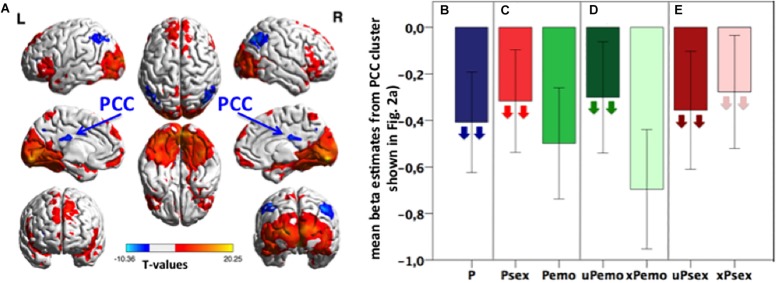
**(A)** Task activation of the contrast *P* > fix. Blue color represents deactivation clusters, red color depicts activation clusters. Displayed with BrainNet Viewer (Xia et al., 2013), *p* < 0.05 FWE, cluster level corrected, with initial threshold *p* < 0.001, *k* size > 191, *n* = 31 subjects. **(B–E)** Bar plots: Extracted mean beta estimates from the PCC correlation cluster (light blue and yellow shown in Figure 2A) for all picture conditions and subconditions versus fixation, showing differential deactivation. Down-pointing arrows in subconditions indicate negative correlation with the Glu/tCr level in dACC. Higher Glu/tCr in the dACC, more deactivation during certain condition is present. Data is presented in mean ± standard error of the mean (SEM), *n* = 31. P, all expected and unexpected emotional and sexual pictures; Psex, expected and unexpected sexual pictures; Pemo, expected and unexpected emotional pictures; uPemo, unexpected emotional pictures; xPemo, expected emotional pictures; uPsex, unexpected sexual pictures; xPsex, expected sexual pictures.

All expected as well as unexpected high salient sexual and low salient emotional pictures (= P) are combined in the “salient picture viewing condition” in this contrast (P versus fix). Picture viewing condition led to significant activation clusters in visual and frontal cortices and significant deactivation in the PCC and bilateral inferior parietal cortices (Figure 1A and Supplementary Table S1a).

#### PCC Deactivation During Salient Picture Viewing Condition Correlated Negatively With the Glu/tCr Concentration in dACC

**FIGURE 2:**
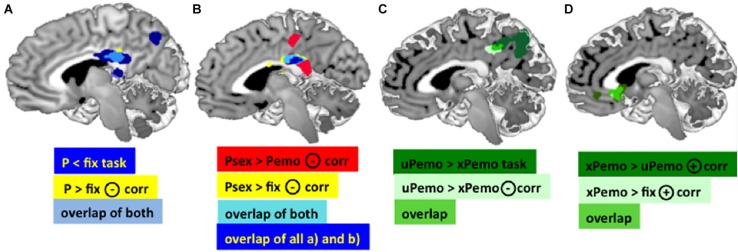
**(A)** Dark blue: Main effect of task in the PCC for contrast *P* < fix. Yellow: Negative correlation between the BOLD response for contrast P > fix in the PCC and Glu/tCr in the dACC. Light blue: Overlap of the PCC clusters, MNI [3 –30 25]. **(B)** Red: BOLD response for contrast Psex > Pemo in the PCC correlates significantly negatively with the dACC Glu/tCr, MNI peak: [2 –28 26]. Yellow: BOLD response for contrast Psex > fix in the PCC correlates significantly negatively with the dACC Glu/tCr, MNI peak: [2 –28 26]. Light blue: overlap of both PCC clusters. Dark blue: overlap of both displayed PCC clusters plus the overlap cluster from **(A)** (light blue and yellow). **(C)** Dark green: Task effect in the PCC/Precuneus in contrast xPemo < uPemo, MNI peak [–14 –52 26]; Light green: Negative correlation of the Glu/tCr in dACC and BOLD response in contrast xPemo < uPemo, MNI peak [–10 –38 34]; Bright green: Overlap of both PCC clusters. **(D)** Overlapping VS clusters. Dark green: Positive correlation of Glu/tCr in dACC and BOLD response for xPemo > uPemo in VS and sgACC MNI peak [2 20 –2]; Light green: Positive correlation of Glu/tCr in dACC and VS BOLD mean estimates for the contrast xPemo > fix, MNI peak: [–2 24 0]; Bright green: overlap of both clusters (note: the light green cluster largely overlaps with the bright green thus is mainly represented in bright green). All pictures are displayed using Mango Software (Lancaster et al.; www.ric.uthscsa.edu/mango), *p* < 0.05 FWE, cluster level corrected, with initial threshold *p* < 0.001 *n* = 27.

The amount of deactivation in the PCC during salient picture viewing condition (all expected and unexpected low salient emotional and high salient sexual pictures against fixation) was significantly negatively correlated with the Glu/tCr concentration in dACC (*p* < 0.05 FWE, cluster level corrected, *k* = 147), Figure 2A, light blue and yellow). Additionally, significant clusters were found for the same correlation in Precuneus, Occipital Cortex, and bilateral Cerebellum (Supplementary Table S1b).

### PCC Deactivation During Picture Viewing Was Salience-Dependent and Modulated by the Glu/tCr Concentration in dACC

The identified PCC cluster (Figure 2A, light blue and yellow, cluster size (*k*) = 2943, MNI: Peak at [16 −40 16]) was used for a *post hoc* ROI analysis to characterize this effect in detail. Mean beta estimates were extracted for different picture subconditions (Figures 1B–E) and correlated with the Glu/tCr concentration in the dACC. Higher Glu/tCr concentration correlated with lower (more negative) betas for all picture conditions (*p* = 0.022, *ρ* = −0.439, *n* = 27, Figure 1B). Within the identified PCC cluster, the deactivations during high salient sexual pictures were smaller (less negative) than for low salient emotional pictures and the deactivation during sexual picture viewing correlated with the Glu/tCr concentration in the dACC (*p* = 0.003, *r* = -0.556, *n* = 27, Figure 1C) while this was not the case for emotional picture viewing (*p* = 0.217, *r* = −0.246, *n* = 27, Figure 1C). Furthermore, deactivations for unexpected emotional pictures were smaller than for expected emotional pictures while only the former correlated negatively with the Glu/tCr level in the dACC (unexpected emotional picture *p* = 0.045, *r* = −0.389, *n* = 27; expected emotional picture *p* = 0.123, *r* = −0.304, *n* = 27, Figure 1D). For these two comparisons, the less deactivating condition was therefore only one significantly correlated with the Glu/tCr concentration in the dACC. A context specificity on glutamatergic influence was, however, not observed for the high salient sexual subconditions, which both correlated significantly with the Glu/tCr level in the dACC (unexpected sexual picture: *p* < 0.001, *r* = −0.687, *n* = 27; expected sexual pictures: *p* = 0.003, *ρ* = −0.549, *n* = 27, Figure 1E).

### Whole-Brain Regression Analysis Confirmed Association Between Salience Perception and the Glu/tCr Level in the dACC

#### Content Specificity: Differences Between High Salient Sexual and Low Salient Emotional Content

**FIGURE 3:**
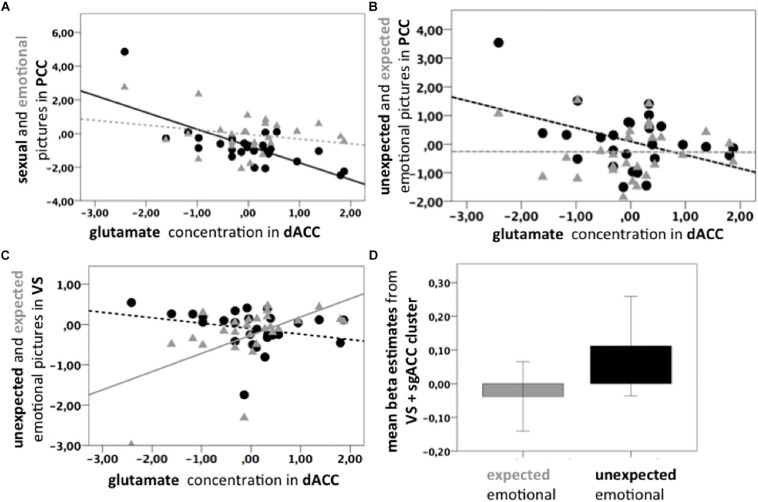
**(A)** Scatter plots. Black line: Significant negative correlation of the Glu/tCr residuals in dACC and PCC BOLD response for Psex > fix (*p* = 0.003, *ρ* = –0.556, *n* = 27); Gray dotted line: Non-significant correlation of Glu/tCr residuals and PCC BOLD response for Pemo > fix (*p* = 0.217, *ρ* = 0.313, *n* = 27). Slopes are significantly different (*p* < 0.001). Beta estimates were extracted from the correlation cluster (light blue and yellow) shown in Figure 2A. **(B)** Black dotted line: Non-significant negative correlation of Glu/tCr residuals in the dACC and PCC BOLD response uPemo > fix (*p* = 0.462, *ρ* = –0.148, *n* = 27). Gray dotted line: Non-significant correlation of Glu/tCr residuals in the dACC and PCC BOLD response for xPemo > fix (*p* = 0.980, *ρ* = 0.183, *n* = 27). Beta estimates were extracted from the correlation cluster (light green and bright green) shown in Figure 2C. **(C)** Gray line: Positive correlation between xPemo > fix and Glu/tCr residuals in dACC (*p* = 0.004, *ρ* = 0.539, *n* = 27). Black dotted line: Non-significant negative correlation between uPemo > fix and Glu/tCr residuals in dACC (*p* = 0.075, *ρ* = –0.348, *n* = 27). Beta estimates were extracted from the bright green correlation cluster shown in Figure 2D. **(D)** Mean beta estimates for xPemo > fix and uPemo > fix represent a relative signal increase for the unexpected condition. Data is presented in mean ± standard error mean (SEM). Beta estimates were extracted from the bright green correlation cluster shown in Figure 2D.

The contrast high salient sexual versus low salient emotional pictures (Psex > Pemo) correlated significantly with the dACC Glu/tCr concentration in dACC in the PCC (*p* < 0.05 FWE, cluster level corrected, with initial threshold *p* < 0.001, *k* = 675, Figure 2B), bilateral Amygdala, left Middle Frontal and Temporal Gyri, left Thalamus, left Inferior Frontal Gyrus, left Cerebellum, right Cuneus, right dorsolateral Prefrontal Gyrus, and brain stem (Supplementary Table S3). PCC correlation cluster of Psex > Pemo contrast overlapped notably with a correlation cluster for Psex > fix (*p* < 0.05 FWE, cluster level corrected, with initial threshold *p* < 0.001, *k* = 600, Figure 2B), whereas no significant whole brain clusters were found for the correlation of Glu/tCr and Pemo > fix. Correlation slopes of the beta estimates for Psex or Pemo > fix (Figure 3A) and residualized Glu/tCr were significantly different (*p* < 0.001, Figure 3A).

#### Glutamate Level in dACC Was Associated With Expectedness Only for Low Salient Emotional but Not for High Salient Sexual Content

Although combined low salient emotional stimuli did not correlate with the Glu/tCr level in dACC on the whole brain level, their respective expected and unexpected subconditions showed significant deactivation difference in the PCC extending to Precuneus (*p* < 0.001, *k* = 1061, Figure 2C, dark green; Supplementary Table 4A), while this difference further correlated with the dACC Glu/tCr (Figure 2C, light green; Supplementary Table 4B). Subjects with higher dACC Glu/tCr had weaker differential responses, and correlation slopes of xPemo and uPemo differed significantly (*p* = 0.002, Figure 3B).

Interestingly, the difference between unexpected and expected low salient emotional pictures showed an additional significant negative correlation with the Glu/tCr level in the dACC for a cluster encompassing the subgenual Anterior Cingulate Cortex (sgACC) and ventral striatum (VS) (*p* < 0.001, *k* = 276), Figure 2D, dark green; for mean beta estimates from VS + sgACC regarding the respective expectancy conditions please see Figure 3D). Here, an overlapping cluster was found for correlation of activations to xPemo > fix (*p* < 0.05 FWE, cluster level corrected, with an initial threshold of *p* < 0.001, *k* = 198, Figure 2D, light green) but not for uPemo > fix. The test for slope differences revealed significant differences between expectedness conditions (*p* < 0.001, xPemo > fix *p* = 0.004, *r* = 0.539, *n* = 27; uPemo > fix *p* = 0.075, *r* = −0.348, *n* = 27; Figure 3C).

Expectedness did not significantly affect the differential amount of activation or deactivation for high salient sexual stimuli, and the whole brain analysis confirmed significant correlation of deactivations to both individual conditions in the PCC (xPsex > fix: *p* < 0.05 FWE, cluster level corrected, *k* = 3398 and uPsex > fix: *p* < 0.05 FWE, cluster level corrected, *k* = 128; Supplementary Table S2).

Additional data check for potential influence of outliers revealed that in the exploratory slope analysis, one subject exceeded 3 IQR for the residualized Glu/tCr values. After removal of that subject, the above-mentioned correlations were all preserved (Supplementary Figure S2).

## Discussion

Here, we showed that the amount of deactivation of the PCC during salient picture viewing in general can be influenced by sexual content as well as expectedness. We interpret this as an indication of attenuation. The amount of attenuation is dependent on the glutamate concentration in the dACC, which correlates negatively with the deactivation of the attenuated picture condition in the PCC. This effect was observed during combined high and low salient picture viewing condition (P), (un)expected high salient sexual (uPsex and xPsex) as well as unexpected low salient emotional picture condition (uPemo). In contrast, this modulation was not observed during the less salient subconditions, combined (un)expected emotional (Pemo) as well as expected emotional (xPemo) picture condition as shown in Figures 1B–E.

Independent from this mechanism in the PCC, an inverse relationship was found in the VS, where the expected emotional pictures correlated with the Glu/tCr level in the dACC leading to a smaller differential activation due to an increased activation of this subcondition. On the other hand, for the same contrast, the differential activity of the expected and unexpected emotional, lower salient stimuli in the PCC extending to the Precuneus correlated with the concentration of the Glu/tCr in dACC but this effect was not clearly driven by isolated effects of expected or unexpected emotional stimuli.

### Modulation of Deactivation by Subjective Salience and Extent of Salience

Our findings align with previous accounts of the DMN deactivation during presentation of visual stimuli. This deactivation by itself is normally considered task-unspecific and regarded as a sign of external orientation of attentional focus (Seeley et al., 2007; Leech and Sharp, 2013). In similar slow event related experimental designs with comparably long fixation and picture durations, the amount of deactivation during presentation of emotional stimuli has been observed to vary as a function of subjective salience (Walter et al., 2008).

By splitting the contrasts of interest into different salience conditions, here we observed differential deactivations in the PCC part of the DMN, which were driven I) by sexual stimuli, showing increasing deactivations (toward levels of emotional stimuli) with higher glutamate concentration in the dACC and II) unexpected emotional stimuli, showing increasing deactivations (toward levels of expected emotional stimuli) with higher glutamate concentration in the dACC.

Our findings indicate that the general (high salient erotic and low salient emotional) salient picture viewing evoked a deactivation in the PCC which correlates with the glutamate concentration in dACC especially during high salient sexual stimuli more than during low salient emotional stimuli perception.

In the same manner, expectancy of salient stimuli in a previous experiment augmented the BOLD response to emotional picture viewing, but not to neutral picture presentation (Bermpohl et al., 2006). This is in accordance with the notion that attentional salience of certain emotional stimuli can be further manipulated by the degree of expectedness. Therefore, our findings on the influence of expectedness on low salient emotional picture viewing conditions may also reflect an effect of salience. One then may assume that the expected emotional content condition is less salient than the unexpected emotional picture condition given different amount of attentional resources to be allocated to the (un)prepared incidence. The lack of differential activity between expected and unexpected sexual stimuli (Supplementary Figure S5) in contrast may then be best interpreted as a ceiling effect induced by the sexual content in terms of salience attribution and attentional refocusing.

### Dependency of Glutamate Concentration in dACC Within Salience Network and Remote DMN

As a main outcome of the study the extent of salience dependent deactivation in the PCC is influenced by the subject’s glutamate level in SN. This effect may be interpreted as a negative effect of (higher) salience processing on the deactivation which can be counterbalanced by a sufficient level of glutamate in the dACC. One interpretation might consider higher levels of glutamate as a representation of higher metabolic activity in the SN and thus an indicator of higher processing efficiency of salient stimuli (Falkenberg et al., 2012). We accordingly derive that the interaction between SN and posterior DMN may work most efficiently when PCC is deactivated while attention toward the stimulus is needed. Higher glutamate concentration in the dACC would then be associated with a more efficient processing ability regarding salience attribution and in turn a stronger posterior DMN deactivation. Lower glutamate levels in the dACC might lead to an altered switching ability between the networks, as seen in various psychiatric diseases (Horn et al., 2010; Falkenberg et al., 2012). That might moreover lead to a prolonged focus toward internal representation of the self-related content, prohibiting the attentional focus to be redirected on the actual source of subjective relevance. This interpretation that assumes that higher self-reference is associated with an increased or prolonged activation of the DMN, which in turn hinders externalized attention is not unchallenged. However, authors such as Northoff et al. (2009) have reported similar effects for stimuli rated with high self-reference. Interestingly, resting state glutamate and GABA concentrations in the PCC were also reported correlating with the PCC deactivation induced by a working memory task. Individuals with high glutamate concentrations in the PCC were associated with a reduced deactivation in the PCC whereas high GABA concentrations in the PCC were linked to an increased deactivation, depending on the cognitive load (Hu et al., 2013). These findings corroborate the assumption that glutamate exhibits a more global effect on BOLD response, whereas GABAergic metabolism might rather influence local BOLD effects as mentioned in the introduction.

The choice of PCC is a consequence of the data, namely the extent of deactivations in our task, rather than one that would be freely set by network considerations. In principle, coactivations would have also been possible to be considered as “network” reflections, and the significant deactivations in bilateral parietal cortex might have been eligible for such an approach. However, as demonstrated by our analysis, lateral components of the DMN do not correlate in their deactivation with SNs MRS (Supplementary Figure S4). Thus, a putatively agnostic hypothesis would have already been rejected. In the same line, it should be acknowledged that the DMN is itself substantially volatile in its contributing components, especially when task designs are considered. This notion is further supported by the fact, that the anterior DMN components were not detected by our whole brain deactivation search analysis. As such, an assumption that the DMN deactivates as a whole and is also affected by the glutamate level in the dACC uniformly, would not find a lot of evidence in light of our data. Moreover, there is a growing body of literature that supports the understanding of a DMN concept as a very coarse account toward true brain behavior and not as a strict definition of regional constituents. While in the specific context of passive resting state, a uniform set of DMN would be more likely, dissipation of effects across e.g., anterior and posterior components is a widely observed fact in the task fMRI research.

It is important to note, that such considerations have to be separated from the actual observation that in the presence of true deactivation, medial and lateral components show a different level of correlation with glutamate level in dACC. This specificity of correlation in medial but not lateral clusters was an interesting observation which may be interpreted as support for an independent interaction of network nodes beyond the organizational principle of their respective modules.

### Variability in Salience Network

Our subsequent analyses specified mechanisms contributing to the effect that (inter-individual) variability in SN leads to differential deactivation. We showed that conditions that are relatively attenuated in their extent of deactivation by salience (intra-individual variability) are particularly affected. Taken together, the influence of SN glutamate on differential activations is mainly driven by the comparably higher salient subcondition such as high salient erotic stimulus.

As an important feature of our experimental design, we could generalize the effects of salience from stimulus inherent salience toward more general salience by presenting pictures at a different degree of expectedness. We found that the amount of deactivation upon unexpected low salient emotional pictures relies on SN glutamate as much as the amount of deactivation upon sexually induced salience. For the same PCC cluster, representing the maximum effect of SN glutamate on picture deactivation in general, both sexual salience and unexpectedness would then be considered sources of higher salience. These sources lead to an attenuation of deactivation – reflective of competition of internal versus external allocation of attentional resources, which, however, becomes less strong for subjects with more efficient SN. Nevertheless, there is a regional variability which needs to be elaborated as an extension of our findings in the PCC and in the VS.

### The Role of the PCC in Attentional Control and the PCC Subregions Showing Different Connectivity and Co-activation Maps

To localize our effects, we identified two different peaks within the PCC. These two peaks belong to the main contrasts high salient sexual content versus low salient emotional contrast (MNI peak: [2 −28 26]) and the unexpected emotional versus expected emotional content [MNI peak: (−10 −38 + 34)]. Moreover, they differ in their respective functional connectivity and co-activation maps (taken from Neurosynth) (Supplementary Figure S3).

According to Neurosynth, the high salient sexual picture contrast cluster [MNI peak: (2 −28 26)] is associated with the dACC. This is in line with the general assumption of the dACC being core region of SN (Supplementary Figure S3a). Contrary, the second PCC peak of the unexpected versus expected low salient emotional contrast cluster (MNI peak: [−10 −38 + 34]) shows co-activation with the pgACC and the anterior DMN (Supplementary Figure S3b). This is in line with the previously reported segregation based on the FC patterns (Margulies et al., 2009): the central part of Precuneus (which can be related to the expectedness cluster) shows FC toward dorsolateral and dorsomedial Prefrontal Cortex, and lateral Inferior Parietal Cortex. This region is associated with integrative processing of cognitive information and higher order executive processing (monitoring of information in working memory, action planning) (Leech and Sharp, 2013).

### Ventral Striatum and sgACC: Expected Emotional Reward Condition

Interestingly, a VS cluster extending to the sgACC was found in an exploratory analysis for the contrast expected versus unexpected low salient emotional content. The expected emotional pictures correlated in their activation with the Glu/tCr level in the dACC leading to a smaller differential activation between the two conditions. Activity changes in the regions associated with reward such as VS have been repeatedly related to motivational processes during sexual stimulation (Hamann et al., 2004; Stark et al., 2005; Abler et al., 2006).

Litt et al. (2010) revealed in an fMRI decision making task that the VS was modulated by both saliency and value signals. The authors observed the PCC as being modulated by value whereas the dACC was modulated by salience computations.

Importantly for the VS/sgACC cluster, we found an opposite effect driving the correlation of SN glutamate and differential activations. In the PCC, it was the more salient condition which was modulated in its amount of deactivation leading to a smaller or larger difference to the less salient and more deactivated condition. In contrast, in the VS/sgACC, the amount of difference was driven by an effect of SN glutamate on the activation of a less salient condition (expected low salient emotional content). Given that the VS/sgACC did also not show a differential modulation of sexual and emotional stimuli by SN glutamate, one may consider the above described modulation of general salience effects to be accounted for mainly in the PCC while the correlation in the VS/sgACC may be rather reflective of a modulation of reward dependent augmentation of neural responses. In such a model, which cannot be fully understood on the basis of our data, particularly in the absence of relevant behavioral information, unexpectedness of a stimulus might induce a novelty bonus (Abler et al., 2012). This is absent for the expected condition and for subjects with higher SN glutamate, this effect could then become smaller due to increased reward related intensity of the stimulus itself (expected emotional content). In a context of (non-pathological) reward processing deficiency, increased SN glutamate would thus characterize those participants who react toward emotional stimuli with intrinsically greater activations and are thus less dependent of stimulation derived from contextual salience (expectedness). While we did not assess measures, which would allow further investigation of this interindividual effect and it was not a primary hypothesis, it is at least stimulating that e.g., Gallinat et al. (2007) found increased sensation seeking behavior in subjects with lower glutamate in dACC. The correlation of glutamate and brain activation in different salient conditions in healthy subjects also imply that some psychiatric symptoms such as attention or internal cue processing might be inherently connected to abnormal metabolite levels (Walter et al., 2009; Horn et al., 2010). Trait reward dependence was further related to increased connections between the dACC and striatum during expectancy of salient stimuli (Li et al., 2017).

## Limitations

Our findings will need to be considered within the natural limitations of the study. Sample size of *n* = 31 for task effects and *n* = 27 for MRS analysis would favor replication in larger sample. For generalizability of our findings one needs to consider that the current findings relied on high validity of the glutamatergic measures which can be separated at high field (Dou et al., 2015). Therefore, translation to other studies will need to be done carefully, particularly if spectroscopic information is derived from lower field strengths. Furthermore, all stimuli used in this paradigm were positive in their content. Next to salience, the dimension of valence is crucial to understanding human brain behavior. Frantzidis et al. (2010) presented an EEG study of valence distinguishing (un)pleasantness and arousal. In the present study our focus was to minimize the impact of valence on the observed correlations. High and low salient stimuli did not differ in valence as they were all positive pleasant stimuli taken from IAPS. However, there is indeed an effect of arousal when contrasting erotic and non-erotic positive emotional IAPS pictures as mentioned in the introduction, e.g., Walter et al. (2008) discussing the relative contributions of different emotional dimensions during viewing of erotic IAPS pictures. Thus, it would be interesting to assess the modulatory effects of glutamate (if any) on the intersection of salience and valence in context of a future study comprising negative stimuli.

We furthermore acknowledge that more recent advances like magnetic resonance spectroscopy imaging (MRSI) offer new possibilities to address multiple MRS measurements in a single study. Instead of choosing a set of single voxels, whole brain acquisition would allow selection of several regional metabolite levels for comparison. However, using 7T acquisition protocol to specify glutamate versus unspecific Glx markers, measured commonly with 3T, such MRSI sequence was not available for ultra high-field MRI.

## Conclusion

This combined fMRI/MRS investigation showed that BOLD response in the PCC upon picture stimulation depends I) on the salience level (sexual content and expectedness) of the stimulus and II) on the individual glutamate level in the SN-related dACC. This study thus indicates relevance of interindividual biochemical variation in a region central to salience processing within a cohort of healthy subjects. Stimulus inherent salience induces an attenuation of the deactivation in the posterior DMN, in the PCC, which can in turn be balanced out by a sufficient level of glutamate in the dACC. In line with prior fMRI/MRS studies, glutamate concentration in the dACC modulates remote DMN activations in the context of salience variability. However, our results show modulation of the PCC rather than whole DMN network or other core regions such as parietal lateral cortex.

Our findings add to previous work on the general effects of metabolites and fMRI activations, that glutamate levels in the SN correlate to the BOLD response during salience perception in the PCC, by shifting the competing attentional resources toward processing of external information and away from processes related to high DMN activity, e.g., self-reference. Subjects with such putatively higher SN efficiency, reflected by higher glutamate levels, would further show less modulation, due to contextual salience in the VS, while the potential interrelation of these two modulations (PCC versus VS) remains unclear.

## Data Availability Statement

All datasets generated for this study are included in the manuscript/Supplementary Files.

## Ethics Statement

The studies involving human participants were reviewed and approved by the institutional review board of Otto von Guericke University Magdeburg. The patients/participants provided their written informed consent to participate in this study.

## Author Contributions

FD, DD, LC, and MW contributed to conception and design of the study. FD and DD organized the database. ML, LC, SL, and LD performed the statistical analysis. FD wrote the first draft of the manuscript. All authors contributed to the manuscript revision, read, and approved the submitted version.

## Conflict of Interest

The authors declare that the research was conducted in the absence of any commercial or financial relationships that could be construed as a potential conflict of interest.

## Footnotes

1 http://www.neuro.uni-jena.de/vbm/

2 http://www.neurobs.com
