# Supplementary material for: Glutamate in Salience Network Predicts BOLD Response in Default Mode Network During Salience Processing
Source: Front Behav Neurosci. 2019 Oct 2;13:232. doi: 10.3389/fnbeh.2019.00232 (PMC6783560; doi:10.3389/fnbeh.2019.00232)
Supplement: Supplementary file 1 [file Table_1.docx]

**10. Supplementary Figures and Tables**

**
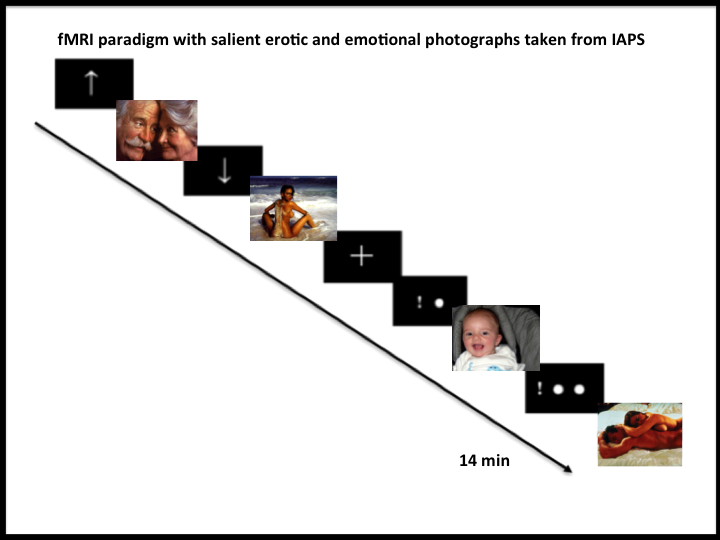
Supplementary Figure 1:** Task fMRI paradigm. Visual cues and exemplary pictures used in the experiment.


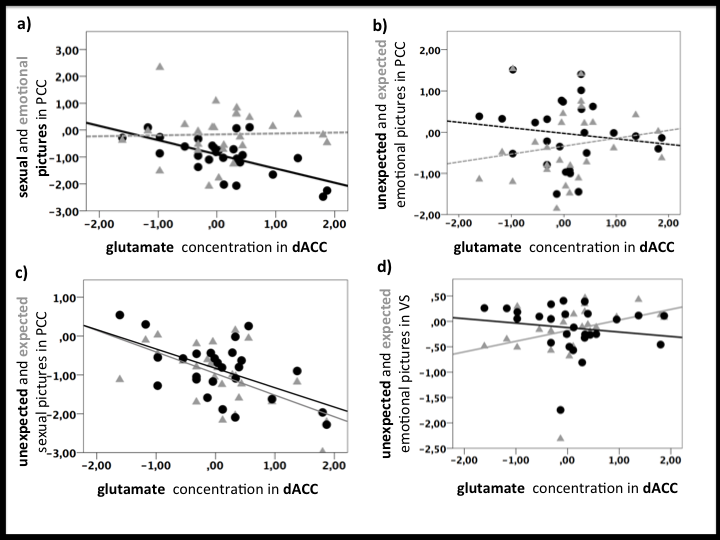


**Supplementary Figure 2: 2a) Black line:** Significant negative correlation of glutamate residuals in dACC and PCC BOLD response for sexual pictures against fixation, *p*= .001, *ρ*= -.621, *n*= 26. **Gray dotted line**: Nonsignificant negative correlation of glutamate residuals in dACC and PCC BOLD response for emotional pictures against fixation, *p*= .886, *r*= .030, *n*= 26. Beta estimates were extracted from the correlation cluster (yellow and light blue) shown in Figure 2a. The test for the slope differences revealed significant difference between conditions (*p*= .004). **2b)** **Black dotted line:** Nonsignificant negative correlation of glutamate residuals in dACC and PCC BOLD response for unexpected emotional pictures against fixation (*p*= .505, *r*= -.137, *n*= 26). **Gray dotted line**: Nonsignificant slightly positive correlation of glutamate residuals in dACC and PCC BOLD response for expected emotional pictures against fixation (*p*= .372, *r*= .183, *n*= 26). Beta estimates were extracted from the correlation cluster (yellow and light blue) shown in Figure 2a. The test for the slope differences revealed significant difference between expectedness conditions (*p*= .003). **2c) Gray line:** Significant negative correlation between expected sexual pictures and dACC Glu residuals (*p*= .002, *r*= -.583 *n*= 26). **Black line**: Significant negative correlation between unexpected sexual pictures and dACC Glu residuals (*p*= .003, *r*= -.557, *n*= 26). Beta estimates were extracted from the light blue and yellow correlation cluster shown in Figure 2a. The test for the slope differences revealed no significant difference between expectedness conditions (*p*= .842). **2d)** **Gray line**: Significant positive correlation between expected emotional pictures and dACC Glu residuals (*p*= .012, *ρ*= .484, *n*= 26). **Black line:** Nonsignificant negative correlation between unexpected emotional pictures and dACC Glu residuals (*p*= .183, *r*= -.270, *n*= 26) Beta estimates were extracted from the yellow and green correlation cluster shown in Figure 2d. The test for the slope differences revealed significant difference between expectedness conditions (*p*= .002).

**
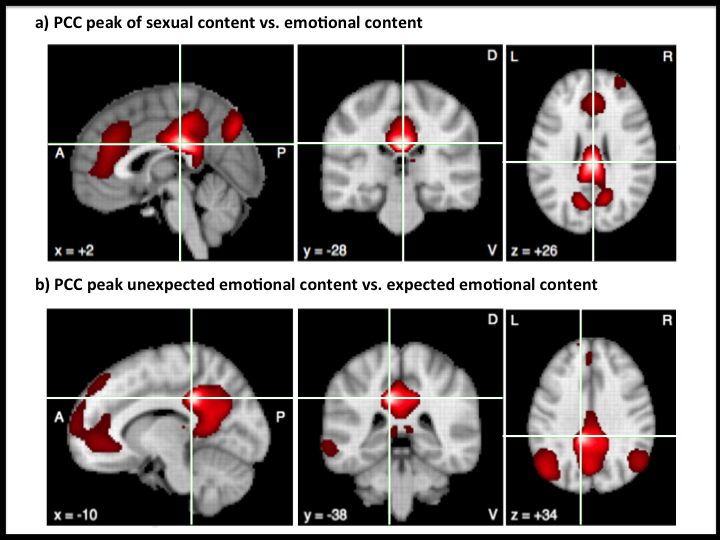
Supplementary Figure 3: 3a)** Functional connectivity and co-activation maps from PCC peak of sexual content vs. emotional contrast taken from Neurosynth (https://neurosynth.org). **3b)** Functional connectivity and co-activation maps from PCC peak of unexpected emotional vs. expected emotional content taken from Neurosynth.org.

**
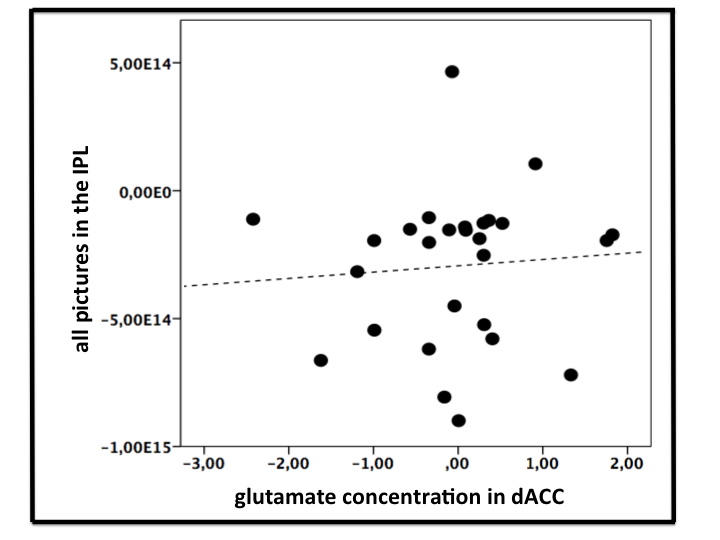
**

**Supplementary Figure 4:** Nonsignificant positive correlation of glutamate residuals in dACC and IPL BOLD response for all pictures (P) versus fixation, *p*= .602, *rho*= .105, *n*= 27.

**Supplementary Table 1:** **a)** Task effects and **b)** correlation with Glu/tCr level in the dACC for contrast salient emotional and sexual pictures versus fixation. Results are reported at p< .05 FWE, cluster level corrected, with initial threshold p< .001.

| **Region** | **X** | **Y** | **Z** | **Cluster size** | **p-value** | **Peak T-value** |
| --- | --- | --- | --- | --- | --- | --- |
| **a) BOLD responses for contrast pictures < fixation: task effect** | | | | | | |
| **1. R Posterior Cingulate Cortex** | **16** | **-40** | **16** | **2943** | **0.000** | **10.36** |
| 1.1 R Posterior Cingulate Cortex | -14 | -42 | 16 |  |  | 9.11 |
| 1.2 R Posterior Cingulate Cortex | 24 | -48 | 14 |  |  | 7.94 |
| **2. R Angular Gyrus** | **46** | **-68** | **46** | **1437** | **0.000** | **9.00** |
| 2.1 R Inferior Parietal Lobule | 46 | -52 | 52 |  |  | 6.94 |
| 2.2 R Angular Gyrus | 54 | -62 | 42 |  |  | 6.89 |
| **3. L Inferior Parietal Lobule** | **-44** | **-58** | **52** | **1430** | **0.000** | **8.70** |
| 3.1 L Inferior Parietal Lobule | -38 | -52 | 44 |  |  | 7.86 |
| 3.2 L Angular Gyrus | -38 | -76 | 46 |  |  | 7.72 |
| **4. R Superior Orbital Gyrus** | **22** | **56** | **-2** | **494** | **0.000** | **6.45** |
| 4.1 R Superior Frontal Gyrus | 24 | 54 | 6 |  |  | 5.86 |
| 4.2 R Superior Frontal Gyrus | 26 | 46 | 2 |  |  |  |
| **5. R Superior Frontal Gyrus** | **22** | **14** | **44** | **156** | **0.040** | **6.10** |
| 5.1 R Superior Frontal Gyrus | 24 | 20 | 54 |  |  | 4.67 |
| 5.2. R Middle Frontal Gyrus | 34 | 12 | 44 |  |  | 3.84 |
| **6. L Middle Frontal Gyrus** | **-32** | **50** | **6** | **467** | **0.000** | **5.90** |
| 6.1 L Middle Frontal Gyrus | -20 | 50 | -4 |  |  | 5.15 |
| 6.2 L Middle Frontal Gyrus | -20 | 40 | -4 |  |  | 4.47 |
| **b) Negative correlation between dACC Glu/tCr level and BOLD responses for contrast pictures > fixation** | | | | | | |
| **1. R Posterior Cingulate Cortex** | **4** | **-30** | **24** | **147** | **0.036** | **5.17** |
| **2. R Occipital Cortex** | **38** | **-88** | **4** | **136** | **0.049** | **5.06** |
| 2.1. R Occipital Cortex | 46 | -80 | 4 |  |  | 4.28 |
| **3. R Cerebelum** | **30** | **-72** | **-26** | **372** | **0.000** | **5.00** |
| 3.1. R Cerebelum | 8 | -74 | -22 |  |  | 4.76 |
| 3.2. R Cerebelum | 24 | -76 | -22 |  |  | 4.68 |
| **4. L Cerebelum** | **-42** | **-60** | **-30** | **233** | **0.004** | **4.96** |
| 4.1. L Cerebelum | -42 | -74 | -22 |  |  | 4.83 |

**Supplementary Table 2:** Negative correlation between the dACC Glu/tCr and BOLD response during expected sexual pictures greater fixation and unexpected sexual pictures greater fixation. Results are reported at p< .05 FWE, cluster level corrected, with initial threshold p< .001.

| **Region** | **X** | **Y** | **Z** | **k** | **p-value** | **T-value** |
| --- | --- | --- | --- | --- | --- | --- |
| **a) Negative correlation with the dACC Glu/tCr for xPsex > fix** | | | | | | |
| **1. L Putamen** | **-22** | **16** | **0** | **992** | **0.000** | **4.83** |
| 1.1 L Insula Lobe | -30 | 16 | 6 |  |  | 5.55 |
| 1.2 L Pallidum | -20 | -8 | 4 |  |  | 4.81 |
| **2. PCC** | **2** | **-28** | **26** | **3398** | **0.000** | **6.52** |
| 2.1. R Pallidum | 18 | 0 | -2 |  |  | 6.28 |
| 2.2. R Cerebelum | 10 | -74 | -26 |  |  | 5.79 |
| **3. L Cerebelum** | **-48** | **-58** | **-28** | **869** | **0.000** | **6.09** |
| 3.1 L Cerebelum | -42 | -76 | -20 |  |  | 6.07 |
| 3.2. L Cerebelum | -46 | -52 | -36 |  |  | 5.43 |
| **4. R IFG p.Opercularis** | **58** | **12** | **8** | **140** | **0.036** | **5.95** |
| **5. R Middle Occipital Gyrus** | **38** | **-88** | **4** | **160** | **0.020** | **5.95** |
| 5.1 R Middle Occipital Gyrus | 50 | -78 | 4 |  |  | 4.31 |
| **6. *R dorsolateral Prefrontal Cortex*** | **10** | **-6** | **64** | **237** | **0.002** | **5.61** |
| 6.1. *R dorsolateral Prefrontal Cortex* | 16 | 10 | 62 |  |  | 4.52 |
| **7. R Middle Frontal Gyrus** | **28** | **14** | **54** | **268** | **0.001** | **5.60** |
| 7.1. R Superior Frontal Gyrus | 26 | 22 | 52 |  |  | 5.27 |
| 7.2. R Superior Frontal Gyrus | 26 | 32 | 50 |  |  | 4.88 |
| **8. L Precuneus** | **-10** | **-72** | **34** | **608** | **0.000** | **5.22** |
| 8.1. R Precuneus | 14 | -72 | 40 |  |  | 4.93 |
| 8.2 R Cuneus | 16 | -68 | 32 |  |  | 4.90 |
| **9.** L Superior Frontal Gyrus | **-14** | **12** | **50** | **497** | **0.000** | **5.15** |
| 9.1. *R posterior medial frontal* | 4 | 6 | 46 |  |  | 5.03 |
| 9.2. L Middle Frontal Gyrus | -24 | 8 | 50 |  |  | 4.64 |
| **b) Negative correlation with the dACC Glu/tCr for uPsex > fix** | | | | | | |
| **1. PCC** | **4** | **-32** | **26** | **128** | **0.032** | **5.55** |
| **2. Brain stem** | **6** | **-24** | **-8** | **122** | **0.040** | **4.56** |
| 2.1. Brain stem | -12 | -18 | -10 |  |  | 4.51 |
| 2.2. R Thalamus | 12 | -22 | -2 |  |  | 3.94 |
| 3. R Cerebelum | 30 | -72 | -24 | 121 | 0.041 | 4.51 |
| 3.1 R Cerebelum | 20 | -76 | -24 |  |  | 4.35 |
| 3.2. R Cerebelum | 12 | -78 | -24 |  |  | 3.91 |

**Supplementary Table 3:** **a)** Task effect and **b)** negative correlation between the dACC Glu/tCr and BOLD activity for the contrast sexual pictures greater emotional pictures. Results are reported at p< .05 FWE, cluster level corrected, with initial threshold p< .001.

| **Region** | X | Y | Z | k | p-value | T-value |
| --- | --- | --- | --- | --- | --- | --- |
| **a) BOLD responses for contrast sexual pictures > emotional picture: task effect** | | | | | | |
| **/** |  |  |  |  |  |  |
| **b) Negative correlation between dACC Glu/tCr and BOLD responses for contrast sexual picture > emotional picture** | | | | | | |
| **1. R Amygdala** | **26** | **-2** | **-14** | **588** | **0.000** | **7.06** |
| 1.2 R Putamen | 30 | 14 | -2 |  |  | 5.17 |
| 1.3. R Insula Lobe | 42 | -6 | 0 |  |  | 5.14 |
| **2. Posterior Cingulate Cortex** | **2** | **-28** | **26** | **675** | **0.000** | **6.80** |
| 2.1. L Posterior Cingulate Cortex | -4 | -40 | 18 |  |  | 5.99 |
| 2.2. R Medial Cingulate Cortex | 6 | -30 | 34 |  |  | 5.22 |
| **3. L Middle Frontal Gyrus** | **-30** | **16** | **32** | **367** | **0.000** | **6.26** |
| 3.1 L Middle Frontal Gyrus | -22 | 8 | 48 |  |  | 5.64 |
| 3.2. L Superior Frontal Gyrus | -16 | 20 | 44 |  |  | 3.87 |
| **4. Left Thalamus** | **-6** | **-8** | **12** |  |  | **6.22** |
| 4.1 R Thalamus | 18 | -8 | 10 |  |  | 5.80 |
| **5. L IFG p.Opercularis** | **-42** | **6** | **10** | **177** | **0.007** | **6.15** |
| **6. R Cuneus** | **14** | **-70** | **32** | **1073** | **0.000** | **6.13** |
| 6.1 L Cuneus | -12 | -64 | 28 |  |  | 5.81 |
| 6.2. R Precuneus | 10 | -66 | 42 |  |  | 5.81 |
| **7. R Dorsolateral Prefrontal Cortex** | **10** | **-12** | **64** | **506** | **0.000** | **5.91** |
| 7.2. R Middle Frontal Gyrus | 26 | 32 | 32 |  |  | 5.01 |
| **8. R Brain stem** | **6** | **-26** | **-28** |  |  | **5.77** |
| 8.1 R Brain stem | 10 | -18 | -26 |  |  | 5.48 |
| 8.2 R Cerebelum | 12 | -36 | -26 |  |  | 5.19 |
| **9. L Cerebelum** | -48 | -58 | -40 | 222 | 0.002 | 5.77 |
| 9.1 L Cerebelum | -32 | -56 | -44 |  |  | 5.19 |
| 9.2 L Cerebelum | -46 | -64 | -48 |  |  | 4.98 |
| **10. L Amygdala** | **-34** | **6** | **-10** | **247** | **0.001** | **5.53** |
| 10.1 L Insula | -40 | -4 | -18 |  |  | 5.03 |
| 10.2. L Amygdala | -24 | 0 | -12 |  |  | 5.01 |
| **11. R Middle Temporal Gyrus** | **54** | **-14** | **-14** | **189** | **0.005** | **5.35** |
| 11.1 R Middle Temporal Gyrus | 46 | -4 | -28 |  |  | 5.14 |
| 11.2 R Middle Temporal Gyrus | 60 | 0 | -14 |  |  | 4.82 |

**Supplementary Table 4: a)** Task effects and **b)** correlation with Glu/tCr level in the dACC for contrast expected emotional pictures versus unexpected emotional pictures. Results are reported at p< .05 FWE, cluster level corrected, with initial threshold p< .001.

| **Region** | **X** | **Y** | **Z** | **k** | **p-value** | **T-value** |
| --- | --- | --- | --- | --- | --- | --- |
| **a) BOLD responses for contrast unexpected emotional pictures > expected emotional pictures: task effect** | | | | | | |
| **1. L Posterior Cingulate Cortex** | **-14** | **-52** | **26** | **1061** | **0.000** | **5.89** |
| 1.1. R Posterior Cingulate Cortex | 8 | -44 | 34 |  |  | 5.82 |
| 1.2 R Posterior Cingulate Cortex | 12 | -46 | 24 |  |  | 5.58 |
| **2. R Angular Gyrus** | **40** | **-70** | **36** | **614** | **0.000** | **5.77** |
| 2.1. R Middle Temporal Gyrus | 44 | -60 | 20 |  |  | 4.73 |
| 2.2. R Angular Gyrus | 34 | -62 | 40 |  |  | 4.51 |
| **3. R Superior Temporal Gyrus** | **60** | **-10** | **0** | **280** | **0.000** | **5.66** |
| 3.1 R Superior Temporal Gyrus | 52 | -18 | 6 |  |  | 4.77 |
| 3.2. R Superior Temporal Gyrus | 62 | -26 | 8 |  |  | 4.66 |
| **4. L Middle Temporal Gyrus** | **-64** | **-24** | **2** | **194** | **0.003** | **4.80** |
| 4.1. L Superior Temporal Gyrus | -56 | -6 | -4 |  |  | 4.68 |
| 4.2. L Middle Temporal Gyrus | -58 | -34 | 4 |  |  | 4.35 |
| **b) Negative correlation between dACC Glu/tCr and BOLD reponses for contrast unexpected emotional pictures > expected emotional picture** | | | | | | |
| **1. Ventral striatum** | 2 | 40 | -2 | 276 | 0.000 | 5.24 |
| 1.1 **R Rectal Gyrus** | 12 | 22 | -14 |  |  | 4.95 |
| 1.2 R Olfactory Gyrus | 4 | 20 | -2 |  |  | 4.95 |
| **2. Posterior Cingulate Cortex** | **-10** | **-38** | **34** | **124** | **0.023** | **5.18** |
| 2.1. Posterior Cingulate Cortex | **0** | **-50** | **36** |  |  | **3.93** |
| 2.2. Posterior Cingulate Cortex | -20 | -38 | 38 |  |  | 3.85 |

**Supplementary Table 5: a)** Task effects and **b)** correlation with Glu/tCr level in the dACC for contrast expected sexual pictures versus unexpected sexual pictures. Results are reported at p< .05 FWE, cluster level corrected, with initial threshold p< .001.

| **Region** | **X** | **Y** | **Z** | **k** | **p-value** | **T-value** |
| --- | --- | --- | --- | --- | --- | --- |
| **a) BOLD responses for contrast unexpected sexual pictures > expected sexual pictures: task effect** | | | | | | |
| **1. R Lingual Gyrus** | **10** | **-70** | **-8** | **859** | **0.000** | **6.34** |
| 1.1 R Lingual Gyrus | 10 | -72 | 2 |  |  | 5.00 |
| 1.2 R Calcarine Gyrus | 18 | -78 | 12 |  |  | 4.74 |
| **2. R Middle Frontal Gyrus** | **28** | **40** | **34** | **430** | **0.000** | **5.27** |
| 2.1. R Middle Frontal Gyrus | 30 | 46 | 26 |  | 0.000 | 5.21 |
| 2.2. R Middle Frontal Gyrus | 30 | 48 | 16 |  |  | 4.13 |
| **3. R SupraMarginal Gyrus** | **60** | **-40** | **34** | **148** | **0.025** | **4.83** |
| 3.1 R SupraMarginal Gyrus | 54 | -48 | 34 |  |  | 4.13 |
| **b) Negative correlation between dACC Glu/tCr for contrast expected sexual pictures > unexpected sexual pictures** | | | | | | |
| **1. R Cerebelum** | **42** | **-56** | **-24** | **206** | **0.003** | **5.52** |
| 1.1 R Inferior Temporal Gyrus | 48 | -52 | -18 |  |  | 4.20 |
| 1.2 R Cerebelum | 40 | -46 | -32 |  |  | 3.81 |
| **2. R ACC (dACC)** | **20** | **-34** | **36** | **338** | **0.000** | **5.22** |
| 2.1. R ACC | 22 | -28 | 46 |  |  | 4.79 |
| 2.2. R ACC | 30 | -24 | 36 |  |  | 4.71 |
| **3. L Cerebelum** | **-44** | **-68** | **-24** | **442** | **0.000** | **5.18** |
| 3.1 L Cerebelum | -38 | -68 | -32 |  |  | 5.03 |
| 3.2. L Inferior Temporal Gyrus | -48 | -56 | -26 |  |  | 4.55 |
